# Comprehensive analysis of the role of immune-related PANoptosis lncRNA model in renal clear cell carcinoma based on RNA transcriptome and single-cell sequencing

**DOI:** 10.32604/or.2023.029563

**Published:** 2023-06-27

**Authors:** WUYAO LIU, CHANGBAO QU, XIAOLU WANG

**Affiliations:** Department of Urology, The Second Hospital of Hebei Medical University, Shijiazhuang, 050000, China

**Keywords:** Programmed cell death, Immune microenvironment, Immunotherapy, Prognostic signature

## Abstract

The high immune infiltration and heterogeneity of the microenvironment in clear cell renal cell carcinoma (ccRCC) result in the variability of prognosis and clinical response. While PANoptosis has strong immunogenicity and is worthy of further study. In this study, data from The Cancer Genome Atlas database was used to obtain immune-related PANoptosis lncRNAs with prognostic value. Subsequently, the role of these lncRNAs in cancer immunity, progression and the therapeutic response was analyzed, and a new prediction model was constructed. Additionally, we further explored the biological value of PANoptosis-related lncRNAs using single-cell data from the Gene Expression Omnibus database. PANoptosis-associated lncRNAs were significantly associated with clinical outcome, immune infiltration, antigen presentation and treatment response in ccRCC. Notably, the risk model, which is based on these immune-related PANoptosis lncRNAs, showed good predictive performance. Subsequent studies on LINC00944 and LINC02611 revealed their high expression in ccRCC and significant correlation with cancer cell migration and invasion. Single-cell sequencing further validated these results and revealed the potential association between LINC00944 and T-cell infiltration and programmed cell death. In conclusion, this study identified the role of immune-related PANoptosis lncRNAs in ccRCC and provided a new risk stratification approach. Furthermore, it highlights the potential of LINC00944 as a prognostic biomarker.

## Introduction

Programmed cell death (PCD), including apoptosis, necroptosis, pyroptosis, and ferroptosis, regulates homeostasis in different ways. With the deepening of research, the regulatory role of PCD in the immune microenvironment along with affecting the occurrence, development, and treatment efficacy of cancer has been confirmed [1]. However, the correlation between the various types of PCD remains unclear. In previous studies, apoptosis, necroptosis, and pyroptosis were found to be activated and regulated simultaneously under certain conditions [2], a phenomenon referred to as PANoptosis, which exhibits a strong correlation with immune responses due to its properties. Based on the immunogenicity of PANoptosis, its role in modulating immune efficacy has been demonstrated [3]. Existing studies on PANoptosis have focused on infectious and autoimmune diseases, while those for tumors are relatively scarce [4,5]. The mechanism of PANoptosis in various tumors, including renal cell carcinoma, warrants further exploration.

Long non-coding RNAs (lncRNAs) are transcripts comprising more than 200 nucleotides [6]. These are widely found in the body and exert various biological functions. Some of these lncRNAs influence progression, prognosis, immune microenvironment, and treatment responses in cancer [7]. Some lncRNAs are closely related to various PCD processes [8–10].

Based on this background, several studies have attempted to investigate the role of PCD-related lncRNAs in cancers. However, research on PANoptosis-related lncRNAs is lacking. Studying the role of PANoptosis-related lncRNAs in tumors is expected to facilitate the understanding of the pathogenesis from a new perspective and explore appropriate treatment strategies.

Clear cell renal cell carcinoma (ccRCC) is a common malignancy of the urinary system [11]. Its high immunogenicity makes immunotherapy one of the main therapeutic methods [12]. However, given its highly heterogeneous tumor microenvironment, the desired effect of immunotherapy for this disease is difficult to achieve [11]. Therefore, it is important to elucidate the regulatory mechanisms underlying the immune microenvironment and identify biomarkers that can better predict the prognosis and treatment effects in patients with ccRCC.

In this study, we comprehensively analyzed the role of immune-related PANoptosis lncRNAs in the progression, prognosis, immune regulation and immunotherapeutic treatment of patients with ccRCC. We also established a risk signature based on these lncRNAs, which aimed at effectively predicting the prognosis and immunotherapeutic responses of these patients. We also evaluated the biological characteristics of the model-related lncRNAs in combination with single-cell data. The findings not only lay a theoretical foundation for a more thorough understanding of the role of PANoptosis-related lncRNAs in ccRCC but also provide a new direction for assessing the specific mechanisms affecting progression and immunotherapeutic effects in these patients.

## Materials and Methods

### Identification and analysis of PANoptosis-related genes

We extracted gene expression and corresponding clinical data from 539 ccRCC tumor samples and 72 normal samples from The Cancer Genome Atlas (TCGA) database (https://portal.gdc.cancer.gov/repository). Gene expression data were standardized to fragment per kilobase million. These data were used as the primary research objects for subsequent weighted correlation network analysis (WGCNA), consistent clustering, model construction and single gene analysis. Additionally, gene expression data from 70 ccRCC samples with complete corresponding clinical information were extracted from the International Cancer Genome Consortium (ICGC) database (https://dcc.icgc.org) for external validation of the risk model. Pyroptosis-, apoptosis- and necroptosis-related gene sets were obtained from previous studies (Suppl. Table S1) [13–15]. Subsequently, we extracted the expression matrix of these gene sets from the TCGA cohort using the limma package in R and screened the genes with significantly differential expressions between tumor and normal tissues. To identify PANoptosis-related genes, we screened those associated significantly with all the three above-mentioned differentially expressed genesets using correlation analysis (*p* < 0.001 and cor > 0.3).

### WGCNA

Immune cell infiltration in TCGA-ccRCC samples was analyzed using the CIBERSORT algorithm [16]. Subsequently, the limma and WGCNA packages in R were employed to cluster the obtained PANoptosis-related genes and elucidate the correlation between gene modules and immune cell infiltration, respectively. CIBERSORT and the limma package were utilized to compare the difference in immune cell infiltration among samples in GSE67501 exhibiting different immunotherapeutic responses. Based on the results obtained, we selected the appropriate gene modules following WGCNA for subsequent analyses.

### Identification of prognostic immune-related PANoptosis-lncRNAs

We used the PERL software to extract lncRNAs from the selected module genes. The expression data of lncRNAs were combined with the survival data of TCGA samples. After excluding samples with incomplete prognostic information, 530 samples were used for subsequent analysis. Thereafter, a univariate COX analysis was performed to obtain prognostic lncRNAs. The differential expression of these prognostic lncRNAs across tissue types was determined.

### Consistent clustering analysis

Based on the expression data of the above prognostic lncRNAs, a consistent clustering analysis was performed and ccRCC samples were divided into two subgroups (Clusters 1 and 2). Differences in prognostic and clinicopathological parameters between the clusters were analyzed using the survival, survminer, and pheatmap packages in R. To elucidate the functions and pathways potentially related to the above clusters, we performed Gene Ontology (GO) and Kyoto Encyclopedia of Genes and Genomes (KEGG) enrichment analyses and Gene Set Enrichment Analysis (GSEA). Genes showing significant differences in their expressions among different clusters were subjected to GO and KEGG enrichment analyses (|logFC| > 1 and FDR < 0.05). Furthermore, the correlation between different clusters and tumor immune cell infiltration was determined. We analyzed and visualized the differences in immune cell infiltration and tumor microenvironment between different clusters. The expressions of some common immune checkpoint genes and antigen-presenting genes among different clusters were assessed.

### Construction and validation of the risk model

The least absolute shrinkage and selection operator (LASSO) regression was used to construct a risk model, and samples with complete prognostic information were divided into training (50%) and test (50%) groups. Next, we classified these samples according to the median risk score and analyzed the prognoses of samples with different risks using the survival and survminer packages. For further validation of the accuracy of this model, receiver operating characteristic (ROC) curves were plotted using the timeROC package. We also plotted risk curves to visualize the survival condition and risk distribution of patients with ccRCC. To determine whether our model could independently predict the outcome of patients with ccRCC, univariate and multivariate regression analyses were performed. Subsequently, a nomogram was established according to the obtained independent prognostic factors and its predictive accuracy and independent prognostic value was determined. The limma and Scatterplot3D packages were used to perform principal component analysis (PCA) for the constructed risk model to determine whether it could well-distinguish ccRCC samples. To assess the applicability of our model, Kaplan–Meier (K-M) analysis was performed for each clinicopathological subgroup. Based on the risk model constructed, the risk score for each sample in the ICGC cohort was calculated. Moreover, the K-M and ROC curves were plotted to assess the model’s efficacy and accuracy in the external cohort, respectively. The log-rank test compared significant differences based on the K-M curves.

### Immune-related analysis

We first identified the functions and pathways enriched significantly in the high- and low-risk groups by GSEA. We downloaded data on infiltrate estimation in all TCGA tumor samples from the TIMER website (http://timer.cistrome.org). We extracted the immune infiltration data of ccRCC predicted using different software and analyzed the correlation between the risk score and immune cell infiltration. We evaluated the association between risk scores and the immune microenvironment based on previously obtained immune microenvironment scoring files. To further assess the differences in immune-related functions between different risk groups, we performed single sample Gene Set Enrichment Analysis (ssGSEA). We calculated the ssGSEA score for immune function in ccRCC samples using limma, GSVA, GSEABase, ggpubr, and reshape2 packages and analyzed the differences in ssGSEA scores between the risk groups. Moreover, genes related to the antigen-presenting machinery were obtained from previous studies and their association with risk scores was determined [17].

### Assessment of the therapeutic mode

We downloaded the Cancer Immunome Atlas (TCIA) scoring file from their website (https://tcia.at/home) to evaluate the responses of ccRCC samples to immunotherapy. Subsequently, we examined the differences in immunotherapeutic efficacies between the risk groups using the ggpubr package in R. We analyzed the association of immune checkpoint genes with risk scores. We calculated the IC50 values (half-maximal inhibitory concentrations) of these agents using the pRRophetic R package to screen suitable therapeutic agents for different risk groups. We analyzed and visualized the differences in IC50 values between high- and low-risk groups using the limma, ggpubr, and ggplot2 packages (*p* < 0.001).

### Validation of the model-obtained lncRNAs in clinical specimens

We collected primary tumor tissue and adjacent normal kidney tissue samples from 10 patients with ccRCC who underwent radical nephrectomy in the Department of Urology, Second Hospital of Hebei Medical University. All patients received no preoperative treatment and were confirmed as ccRCC through postoperative pathology. The experimental protocol was approved by The Ethics Committee of the Second Hospital of Hebei Medical University (2022-R126), and written consent was obtained from each participating patient. Total RNA from these specimens was isolated using Trizol (Invitrogen, CA, USA). Reverse transcription was performed using the MonScript^™^ RTIII Super Mix with dsDNase (Mona, Suzhou, China). Quantitative real-time polymerase chain reaction (qRT-PCR) was performed on the Bio-Rad CFX96 system (Bio-Rad, CA, USA). Glyceraldehyde-3-phosphate dehydrogenase was used as an internal reference. Relative lncRNA expressions were estimated using the 2^−ΔΔCt^ method. The sequences of primers used herein are listed in Suppl. Table S2. The Prism 9 software (version 9.5.0) was used to visualize the results.

### Cell lines and cell culture

The ccRCC cell lines 786-O, Caki-1 and normal renal tubular epithelial cell HK-2 were obtained from the American Type Culture Collection (VA, USA). These cells were cultured in Dulbecco’s Modified Eagle’s Medium (DMEM) (Invitrogen, Grand Island, NY, USA) containing 10% Fetal Bovine Serum (FBS) (Clark Bio, Claymont, DE, USA) and 1% penicillin/streptomycin (Solarbio, Beijing) at 37°C with 5% CO_2_.

### Cell transfection

To further examine the biological properties of PANoptosis-related lncRNAs, we selected two model-associated lncRNAs with the highest risk coefficients (LINC00944 and LINC02611) for knockout assays. Corresponding small interfering RNA (si-RNA) constructs for each of these lncRNAs (GenePharma, Shanghai, China) were transfected into 786-O and Caki-1 cells using the Lipofectamine 2000 reagent (Invitrogen) following the manufacturer’s instructions. Transfected cells were incubated for another 24 h. The sequence of siRNA constructs is shown in Suppl. Table S3.

### Wound healing assay

We seeded 2 * 10^5^ tumor cells into each well of a six-well plate. After cell adhesion and transfection, a 10 μl pipette tip was used to scratch the cells gently along a straight line. The cells were cultured for 24 h after changing the FBS-free media. The experiment was repeated thrice. ImageJ software was utilized to measure the average width of scratches.

### Transwell assay

We used a 24-well Transwell chamber (Millipore, MA, USA) with a pore size of 8 μm to assess cell invasion potential. The transfected tumor cells were resuspended in an FBS-free medium, and the cell suspension containing 1 * 10^5^ cells was added to the upper chamber containing Matrigel (BD Biosciences, NJ, USA). Subsequently, 750 μl of medium with 10% serum was added to the lower chamber, and the cells were incubated for another 12 h. After formaldehyde fixation and crystal violet staining, cells on the lower surface of the filter membrane were counted and imaged under a microscope.

### Preliminary exploration of the biological characteristics of the model-obtained lncRNAs

We first detected the expressions of LINC00944 and LINC02611 in 786-O, Caki-1 and HK-2 cells using qRT-PCR. Next, TCGA data was used to examine the association of these lncRNAs with immune cell infiltration and expressions of the immune checkpoint, antigen-presenting, ferroptosis and cuproptosis genes. Ferroptosis and cuproptosis-related genes were extracted from previous studies [18,19]. Moreover, the differences in TCIA scores between the high- and low-expression groups for LINC00944 or LINC02611 were compared using the ggpubr package in R. Results of the immune-related scores and GSEA were obtained from the CAMOIP website (http://220.189.241.246:13838/#shiny-tab-home).

### Single-cell analysis

Single-cell sequencing data of ccRCC were obtained from the GEO database (GSE156632). Subsequently, the Seurat R package was used to process the data, and cells with gene numbers between 200 and 4500 and mitochondrial gene percentages below 10% were screened for subsequent analysis. After normalizing the above data, PCA dimension reduction and clustering (dims = 1:30 and resolution = 0.6) were performed. The harmony package was used to eliminate batch effects between samples. Furthermore, the irGSEA package was used for gene set scoring.

## Results

### Identification of PANoptosis-related gene modules associated with immune cell infiltration

**Figure 1:**
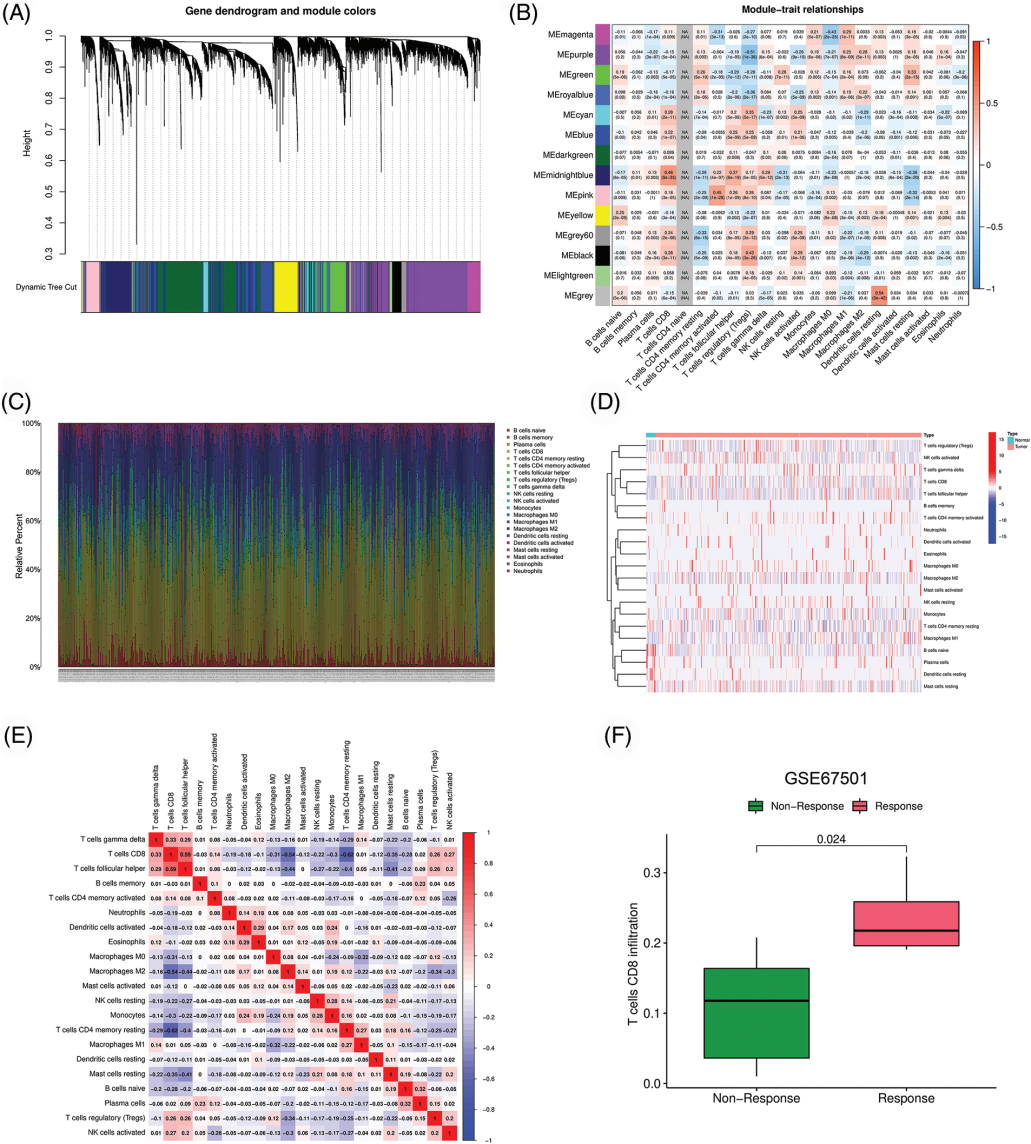
Identification of immune-associated PANoptosis genes. (A) Dendrogram of generation of different co-expression modules. (B) Heatmap of correlation between gene modules and immune cell infiltration. (C–E) Characteristics of immune cell infiltration in TCGA-ccRCC samples. (F) Difference of CD8 T cell infiltration among different response groups to PD-1 treatment (GSE67501).

**Figure S1:**
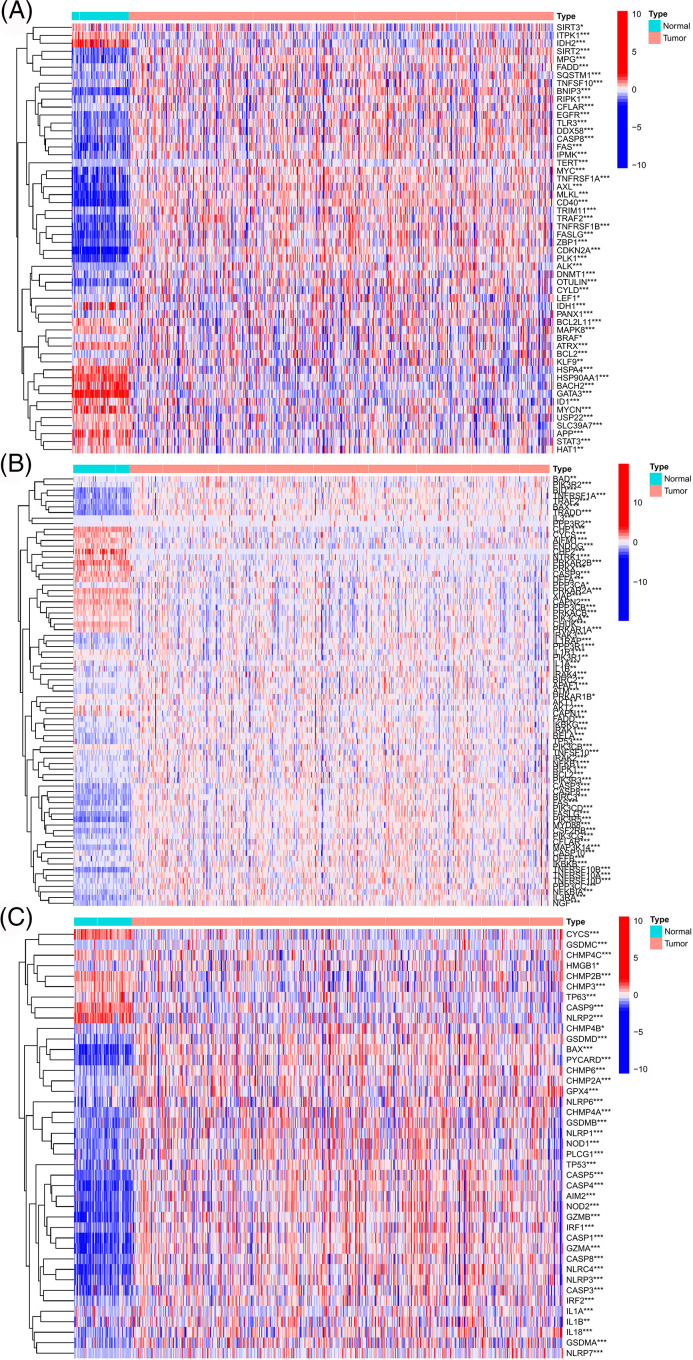
The genes with significant expression differences between TCGA-ccRCC tumor and normal tissues were screened from the necroptosis-related gene set (A), apoptosis-related gene set (B), and pyroptosis-related gene set (C), respectively.

We obtained the transcriptome data of 539 ccRCC samples and 72 normal samples from the TCGA database. Gene with differential expression between cancer and para-cancer tissues were screened from the pyroptosis-, apoptosis- and necroptosis-related gene sets (Suppl. Fig. S1). Subsequently, correlation analysis using the above three gene sets identified 15,378 PANoptosis-related genes. Owing to the strong correlation between PANoptosis and tumor immunity, we performed WGCNA to examine the association between PANoptosis-related genes and immune cell infiltration (Figs. 1A and 1B). The immune landscape in the TCGA-ccRCC cohort is shown in Figs. 1C–1E. The infiltration level of CD8 T cells was higher in ccRCC tumor tissues than in normal tissues. Moreover, CD8 T cells have been reported to be associated with prognoses and immunotherapeutic responses of patients with ccRCC [20]. Consistent results were obtained from our analysis of the GSE67501 dataset. Patients with higher CD8 T cell infiltration had better immunotherapeutic responses (Fig. 1F). Based on the above results, the midnight blue gene module significantly associated with CD8 T cells was selected for subsequent analyses.

### Identification of prognostic immune-associated PANoptosis lncRNAs

**Figure 2:**
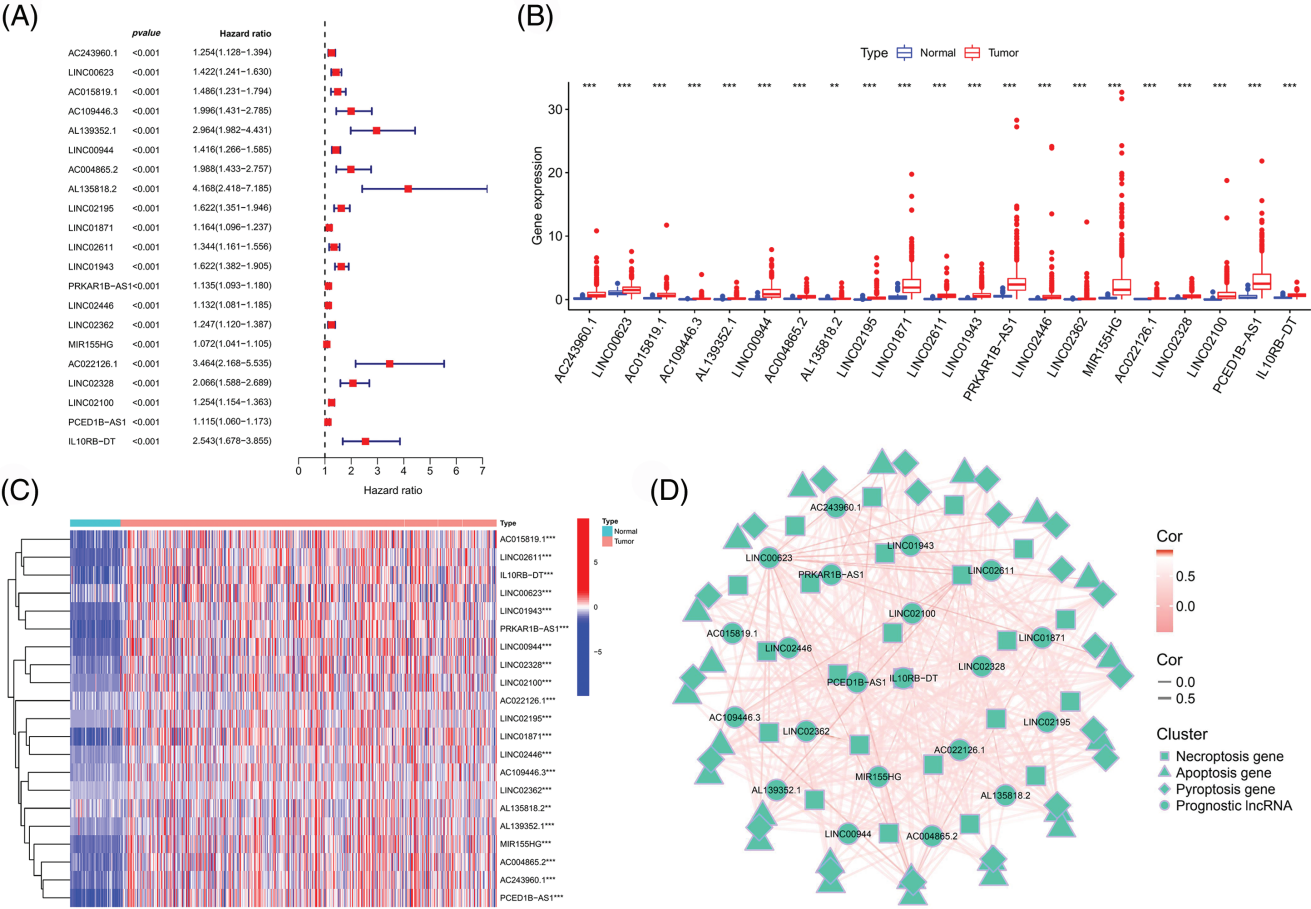
Identification of prognostic immune-associated PANoptosis lncRNAs. (A) Forest plot of prognosis-related lncRNAs. Boxplot (B) and heatmap (C) of the differential expression of prognostic lncRNAs in different tissue types. (D) Network map of the correlation between prognostic lncRNAs and PANoptosis-related genes.

LncRNAs were extracted from the midnight blue gene module. Subsequently, we combined the expression matrix of these lncRNAs with survival data and obtained the prognostic lncRNAs using univariate Cox regression analysis (Fig. 2A). The differential expressions of the prognostic lncRNAs in the TCGA-ccRCC tumor *vs*. normal tissues are shown in Figs. 2B and 2C. These lncRNAs were highly expressed in tumor tissues and associated with a poor prognosis. The correlation between these lncRNAs and PANoptosis-related gene sets is shown in Fig. 2D.

### Consistent clustering analysis for PANoptosis lncRNAs

**Figure 3:**
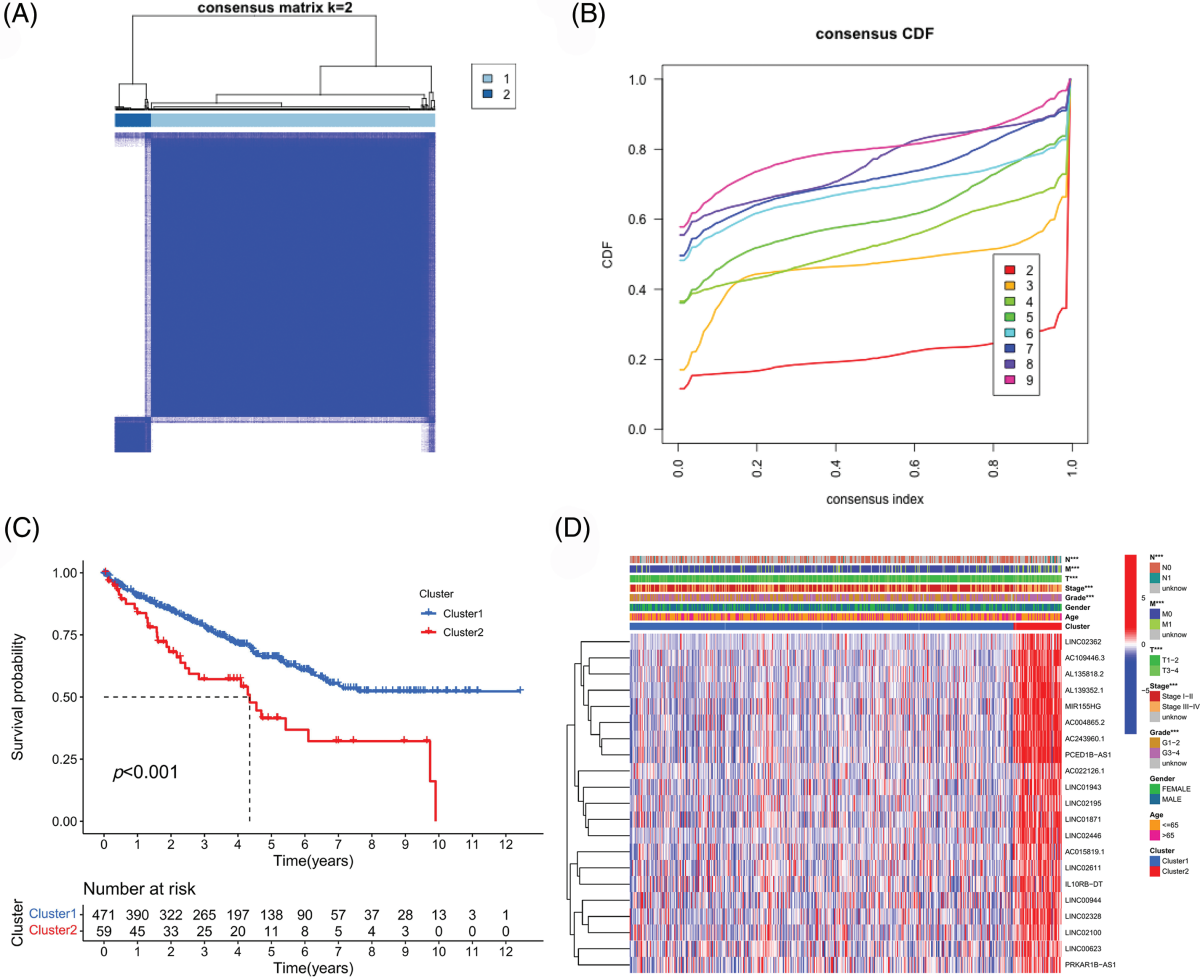
Consistency cluster analysis. (A) Consistency clustering divided TCGA-ccRCC samples into two clusters. (B) Consensus clustering cumulative distribution functions (CDF) for k = 2 to 9. (C) Survival curves of different clusters. (D) Correlation heatmap between clinicopathologic features and clusters.

Consistent clustering analysis was performed based on the expression data of selected prognostic PANoptosis-related lncRNAs and we divided the TCGA-ccRCC samples into two clusters (Figs. 3A and 3B). Cluster 2 was found to show a worse prognosis overall than cluster 1 (Fig. 3C). Moreover, cluster 2 was associated with poor clinicopathological features (higher pathological grade and more advanced disease stage; Fig. 3D).

**Figure 4:**
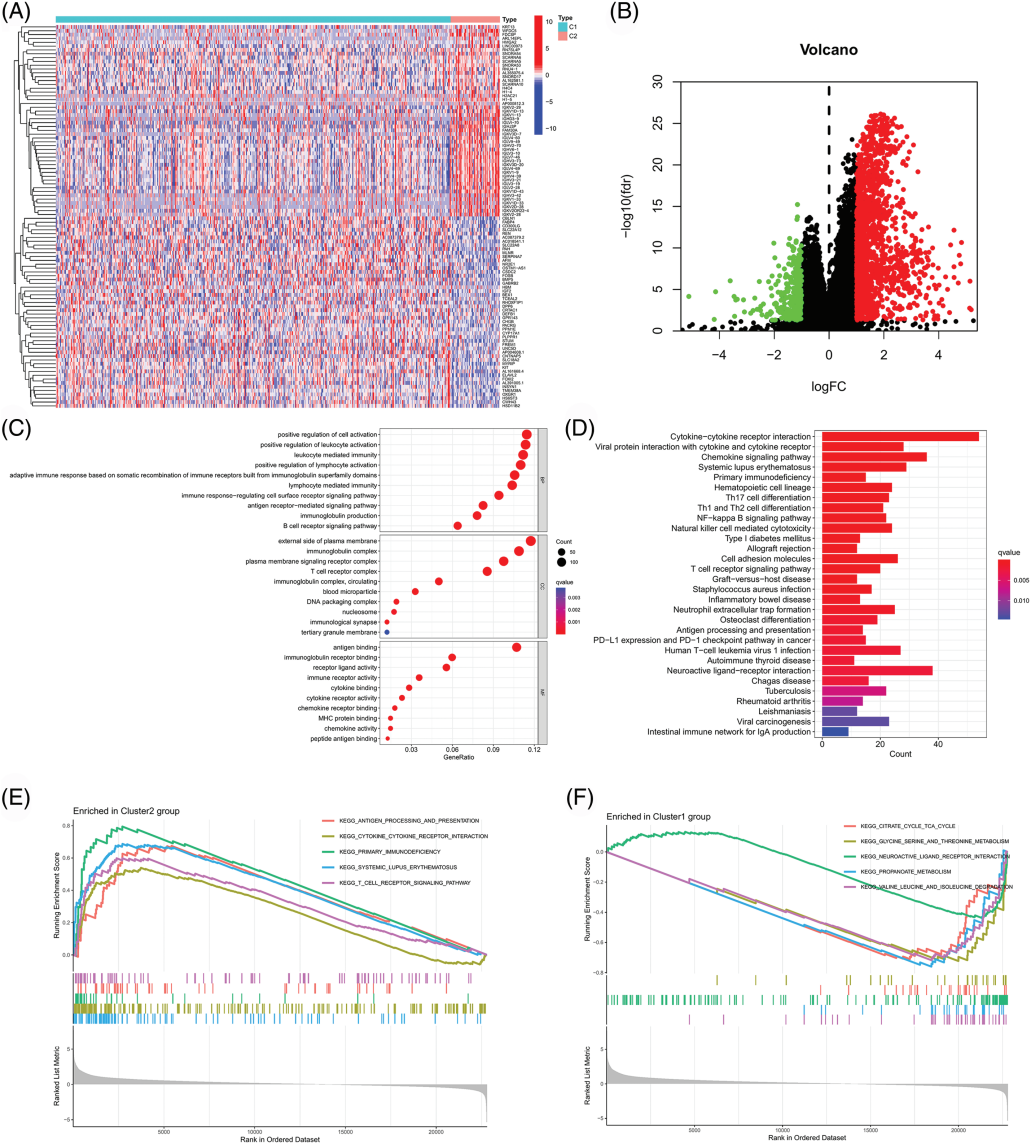
Functional enrichment analysis of different clusters. Heatmap (A) and volcano map (B) of differentially expressed genes among different clusters. (C) Bubble chart for GO enrichment analysis. (D) Bar chart for KEGG enrichment analysis. (E, F) GSEA in different clusters.

To further explore the mechanism underlying the above results, we first screened genes that were differentially expressed across clusters (logFC > 1 and FDR < 0.05). Subsequently, GO and KEGG enrichment analyses and GSEA were performed for these genes, and these results are shown in Fig. 4. These genes were mainly related to immune responses like antigen binding, cytokine activity, chemokine activity, T cell receptor complex, antigen receptor-mediated signaling pathway, chemokine signaling pathway, cytokine-cytokine receptor interaction, antigen processing and presentation, PDL1 expression, PD1 checkpoint pathway in cancer, etc. These findings also suggested a strong association between PANoptosis-related lncRNAs and tumor immunity. These enriched immune functions and pathways may be associated with cancer progression.

**Figure 5:**
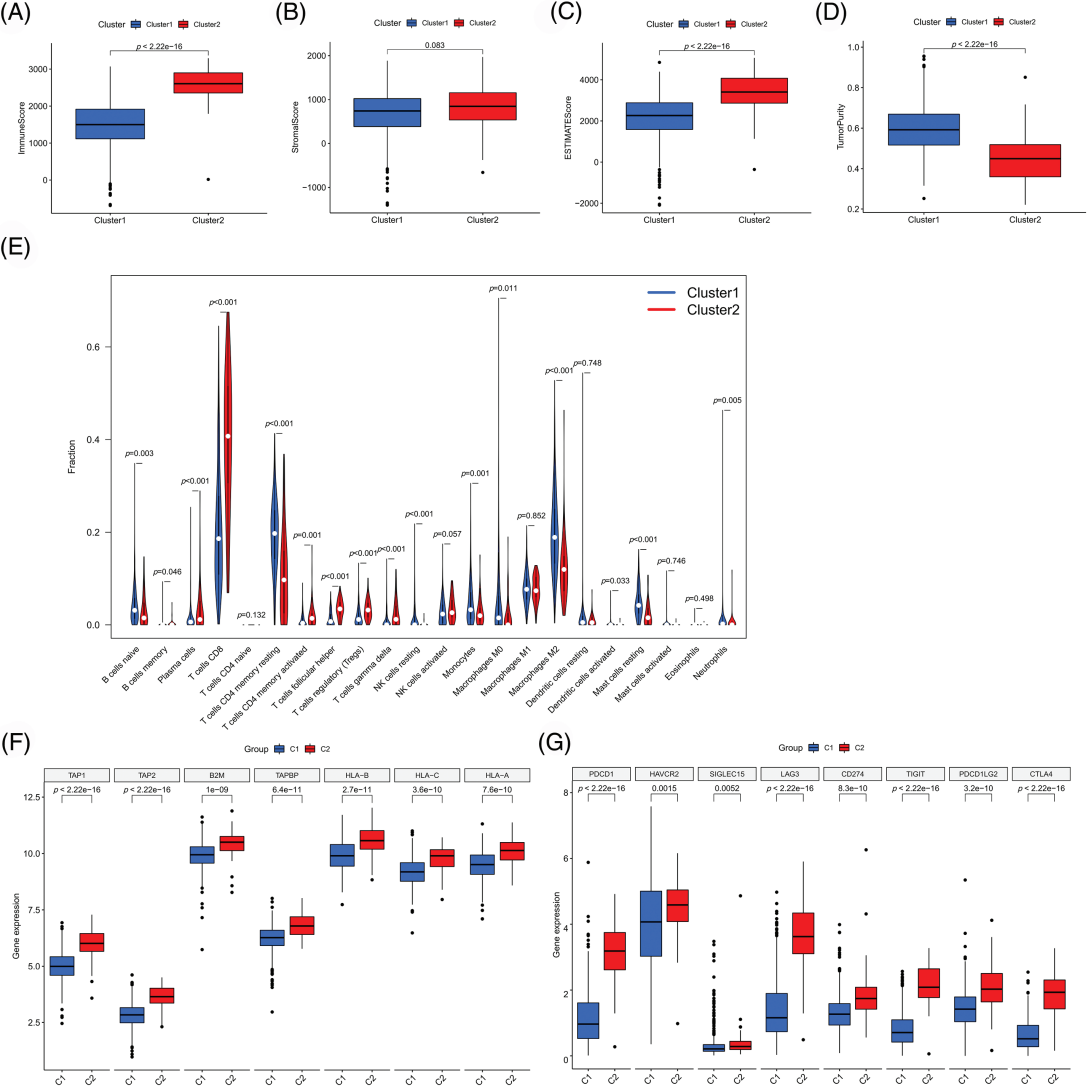
Immune-related analyses among different clusters. Differences in immune microenvironment scores (A–D) and immune cell infiltration (E) among different clusters. Differences in expression of antigen presentation genes (F) and immune checkpoint genes (G) among different clusters. *p < 0.05, **p < 0.01, and ***p < 0.001.

To validate our results, we examined the differences in immune cell infiltration and immune microenvironment between the clusters (Figs. 5A–5E). Compared to cluster 1, cluster 2 exhibited higher immune cell infiltration, lower tumor purity and higher antigen-presenting gene expression (Fig. 5F), which were consistent with previous results. Moreover, the selected PANoptosis-related lncRNAs were associated with CD8 T cell infiltration. Notably, CD8 T cell infiltration has been reported to be associated with immunotherapeutic responses. To further examine the effect of PANoptosis-related lncRNAs on treatment with immune checkpoint inhibitors, we analyzed the differential expression of immune checkpoints between different clusters. As shown in Fig. 5G, the expression of immune checkpoints, including PD1 and CTLA4, was higher in cluster 2 than in cluster 1, highlighting the influence of PANoptosis-related lncRNAs on immunotherapy.

### Construction and validation of the risk model

**Figure 6:**
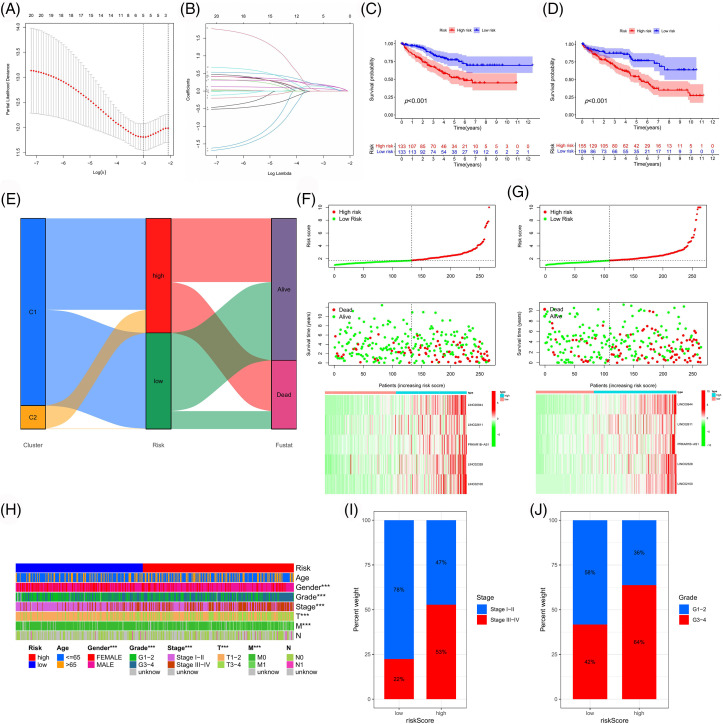
Construction and validation of the risk model. (A, B) Using LASSO regression analysis, we developed a risk model based on five PANoptosis-associated lncRNAs. Survival curves of the risk model in the training (C) and test (D) cohort. (E) Sankey diagram of the association between different classifications and clinical outcomes of TCGA-ccRCC samples. Risk curves of the risk model in the training (F) and test (G) cohort. (H) Correlation heatmap between clinicopathologic features and risk subgroups. (I, J) Percentage bar plots of clinicopathological parameters in different risk subgroups.

Using LASSO regression analysis, we screened LINC00944, LINC02611, PRKAR1B-AS1, LINC02328 and LINC02100 from 21 prognosis-related PANoptosis lncRNAs (Fig. 2B) and constructed the risk model (Figs. 6A and 6B). TCGA-ccRCC patient data was used for model construction and evaluation. The risk score for each TCGA-ccRCC sample was calculated based on the expression and risk coefficient of these lncRNAs as follows: risk score = 0.1835 × LINC00944 + 0.1681 × LINC02611 + 0.0631 × PRKAR1B-AS1 + 0.1236 × LINC02328 + 0.1563 × LINC02100. The model classified the TCGA-ccRCC samples into two groups with significantly different prognoses (Figs. 6C and 6D). High-risk samples exhibited poorer outcomes (Figs. 6E–6G) and were associated with worse clinicopathological features than low-risk samples (Figs. 6H–6J).

**Figure 7:**
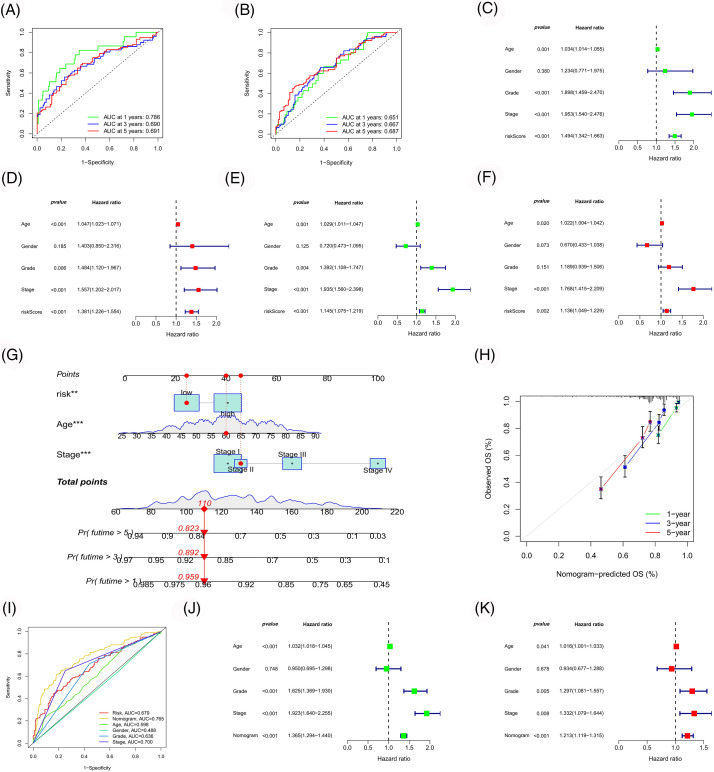
Model evaluation and nomogram construction. ROC curves of the risk model in the training (A) and test (B) cohort. Univariate (C, E) and multivariate (D, F) Cox analyses of the risk model in the training (C, D) and test (E, F) cohort. (G) Construction of a nomogram. Calibration curve (H) and ROC curve (I) of the nomogram. Univariate (J) and multivariate (K) Cox analyses of the nomogram.

Subsequently, the ROC curves for the model were plotted (Figs. 7A and 7B). The 1-, 3- and 5-year ROCs for the training and test cohorts were 0.786, 0.690 and 0.691 and 0.651, 0.667 and 0.687, respectively, indicating a high predictive accuracy for the model. Additionally, the independent prognostic value of our model was evaluated (Figs. 7C–7F), identifying three independent prognostic factors (age, risk score and stage). Following this, a nomogram was constructed with higher predictive accuracy than other clinicopathological features (Figs. 7G and 7H). Moreover, it can independently predict the prognosis of patients with ccRCC (Figs. 7I–7K).

**Figure 8:**
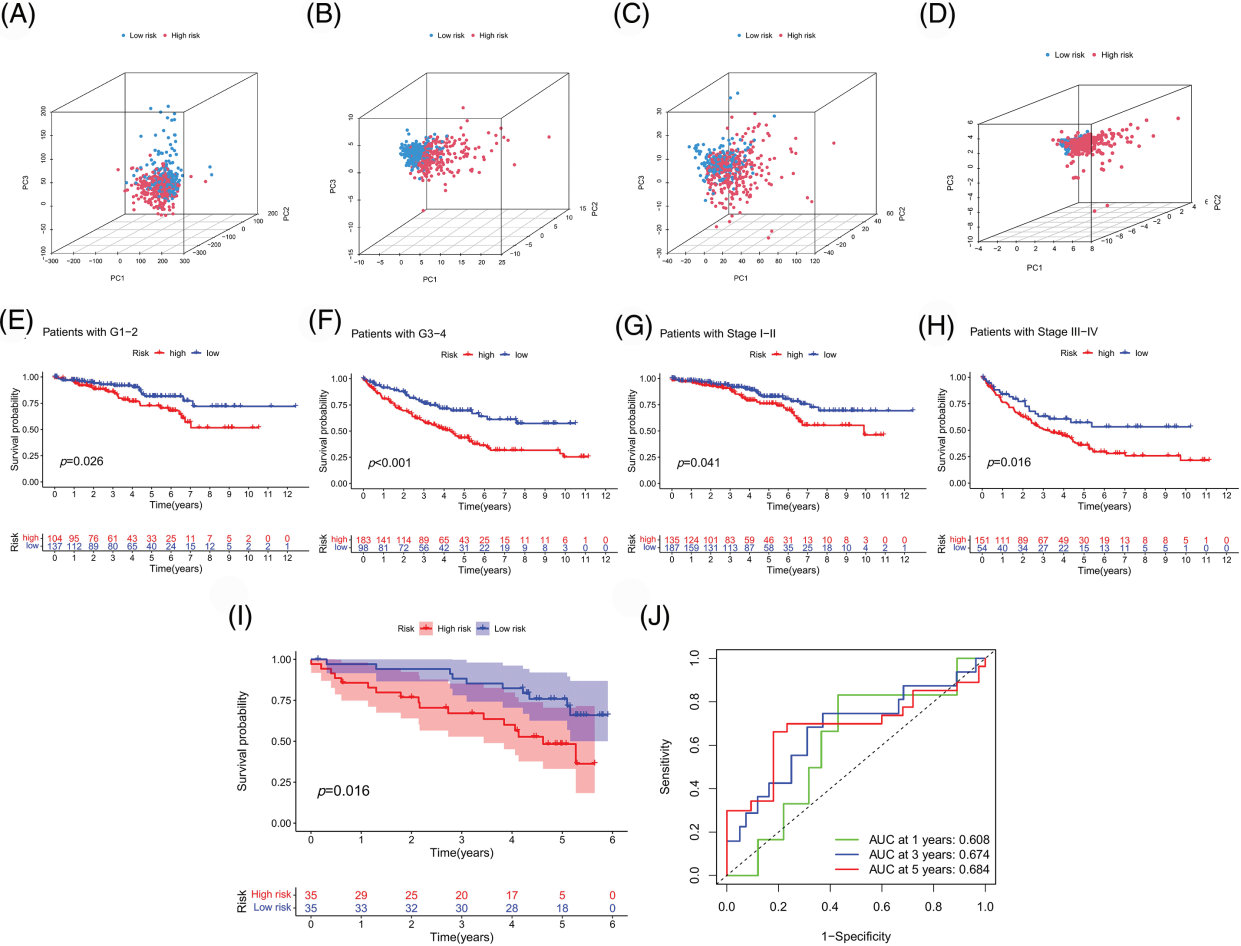
Assessment and validation of the risk model. PCA based on whole genes (A), PANoptosis-associated genes (B), PANoptosis-associated lncRNAs (C), and model-associated lncRNAs (D). (E–H) Survival analyses of the model were performed according to different clinical features. Survival (I) and ROC (J) curves were plotted to validate our model in the ICGC cohort.

To further evaluate the applicability and sample partitioning ability of our model, PCA and K-M analysis were conducted (Fig. 8). Compared to other gene sets, this model could better distinguish between high- and low-risk samples (Figs. 8A–8D). Our model could also be applied to samples with different clinical characteristics (Figs. 8E–8H). Finally, we validated the predictive performance of the risk model using the ICGC cohort, wherein patients with different prognoses were well-classified according to the risk score, with a high prediction accuracy (Figs. 8I and 8J).

### Immune-related analysis

**Figure 9:**
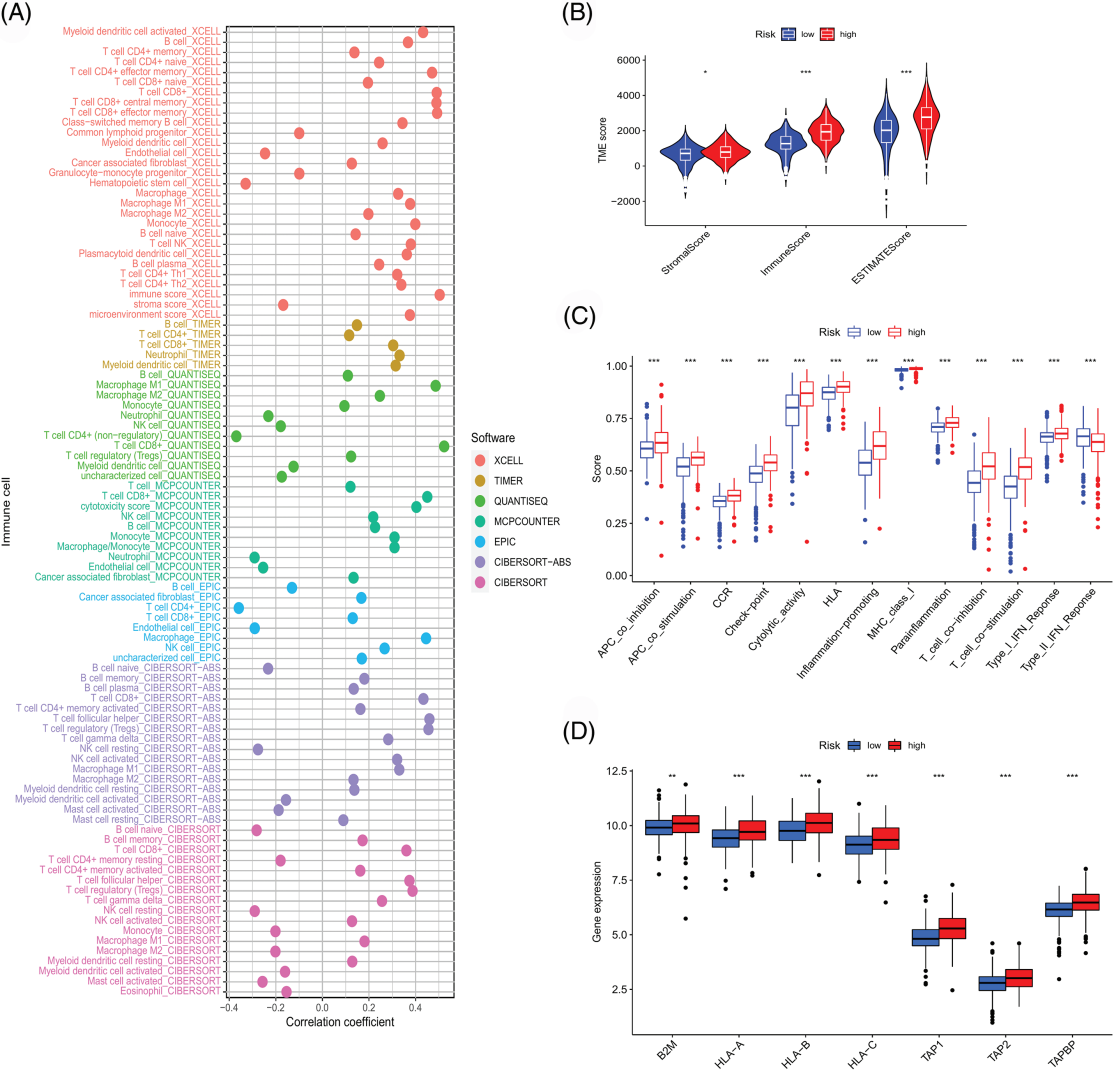
Immune-related analyses. (A) Correlation between risk score and immune cell infiltration obtained by different software. Differences in immune microenvironment score (B), immune-related pathway (C), and antigen presentation gene expression (D) among risk groups.

**Figure S2:**
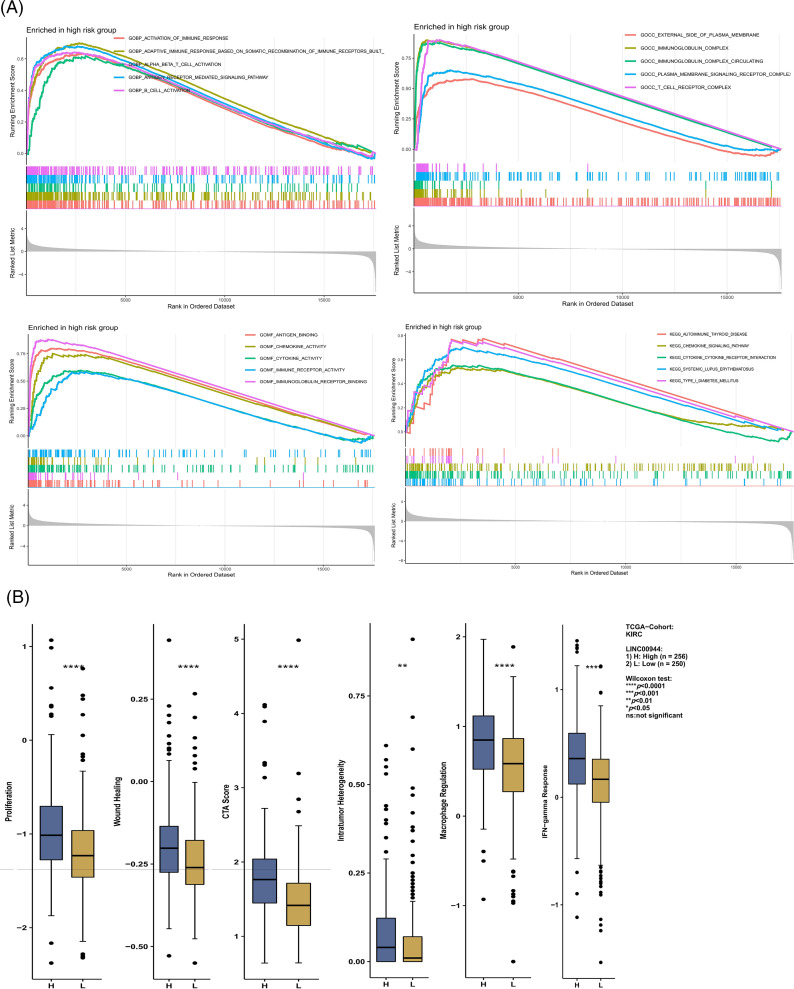
Enrichment analysis and immune-related analysis. (A) Enrichment analysis based on risk score. (B) Differences in immune-related scores between high and low expression groups of LINC00944.

Through GSEA, functions and pathways enriched in the high-risk group of the TCGA cohort were observed to be mainly related to immunity (Suppl. Fig. S2A). To confirm this association, a series of immune-related analyses using the TCGA sample data was performed. We assessed immune cell infiltration in TCGA-ccRCC samples and examined its correlation with risk scores (Fig. 9A). The risk score correlated positively with CD8 T cell infiltration, which was consistent with the results of consistent clustering analysis and WGCNA. Moreover, the risk score was also significantly associated with the immune scores of the microenvironment and extensive activation of immune-related pathways (Figs. 9B and 9C). MHC class I molecules can activate CD8 T cells through antigen presentation [21]. Thus, we further examined the effect of antigen presentation on cancer progression. Several antigen presentation-related genes were selected and their correlation with risk scores was analyzed. These genes were significantly upregulated in the high-risk group (Fig. 9D).

### Assessment of therapeutic modes

**Figure 10:**
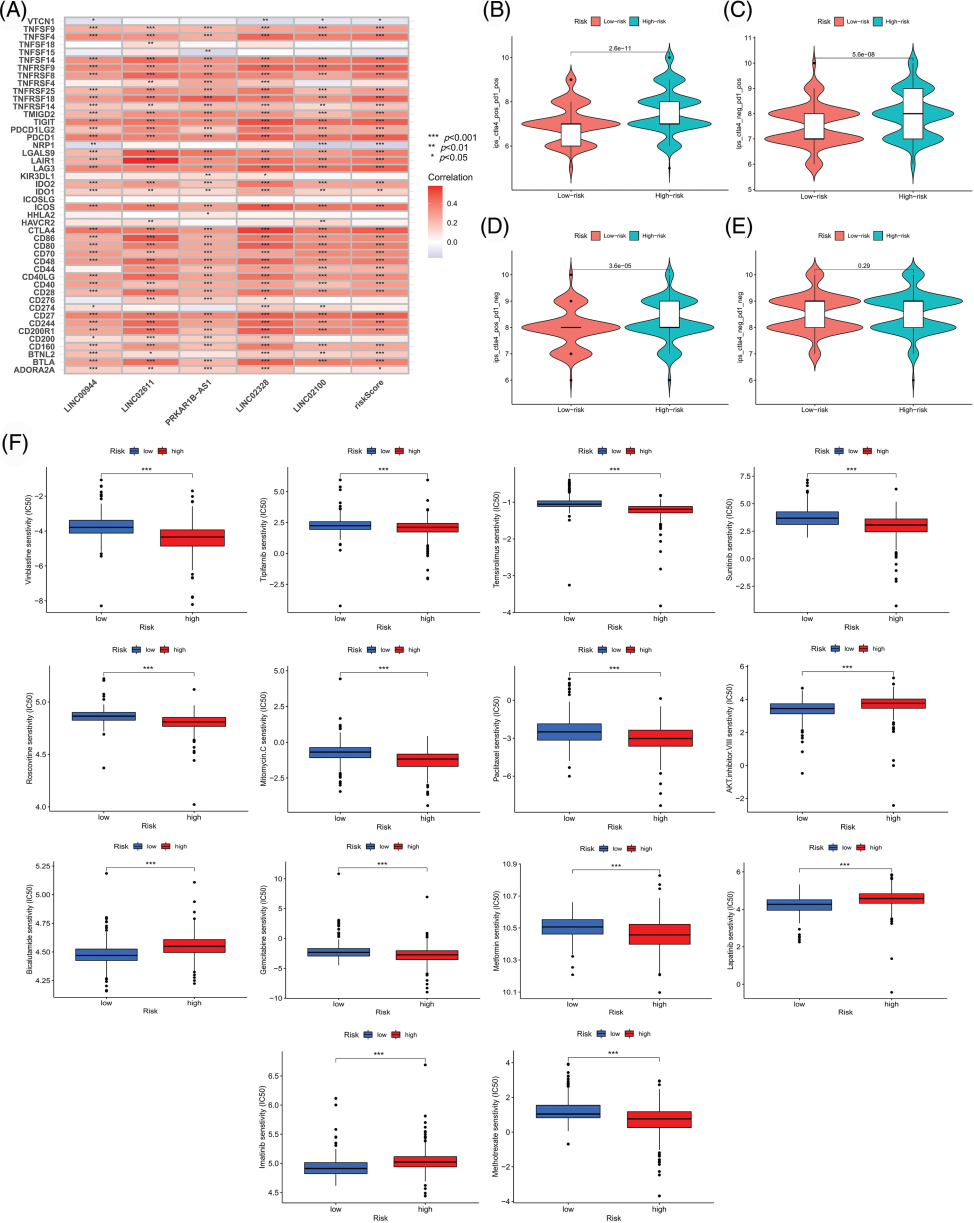
Exploration of therapeutic mode. (A) Association of immune checkpoint genes with our risk model. Differences in response between risk groups when receiving combined anti-CTLA4 and anti-PD-1 therapy (B) and when receiving anti-PD-1 (C) or anti-CTLA4 (D) therapy alone. (E) Differences in response between risk groups without receiving anti-CTLA4 and anti-PD-1 therapy. (F) Differences in drug susceptibility among risk groups.

We used the TCGA sample data to assess the clinical significance of our model in ccRCC treatment. We first analyzed the correlation between risk scores and immune checkpoint genes. As shown in Fig. 10A, the expressions of immune checkpoints, including CTLA4 and PD1, were significantly positively correlated with the risk score, suggesting that the patients with high risk might benefit more from immune checkpoint inhibitor therapy. Subsequently, we evaluated the differences in immune responses between the high- and low-risk subgroups by analyzing the TCIA scores. High-risk samples who received anti-PD1 therapy, anti-CTLA4 therapy or a combination of both showed a better immune response than low-risk samples (Figs. 10B–10E). Moreover, several drugs suitable for patients according to the risk stratification were identified. High-risk samples were more sensitive to drugs like vinblastine, temsirolimus, sunitinib, roscovitine, gemcitabine, metformin, methotrexate, mitomycin C, tipifarnib and paclitaxel. Low-risk samples were more likely to benefit from treatment with drugs like AKT inhibitor VIII, bicalutamide, imatinib and lapatinib (Fig. 10F).

### Validation of the model-related lncRNAs

**Figure 11:**
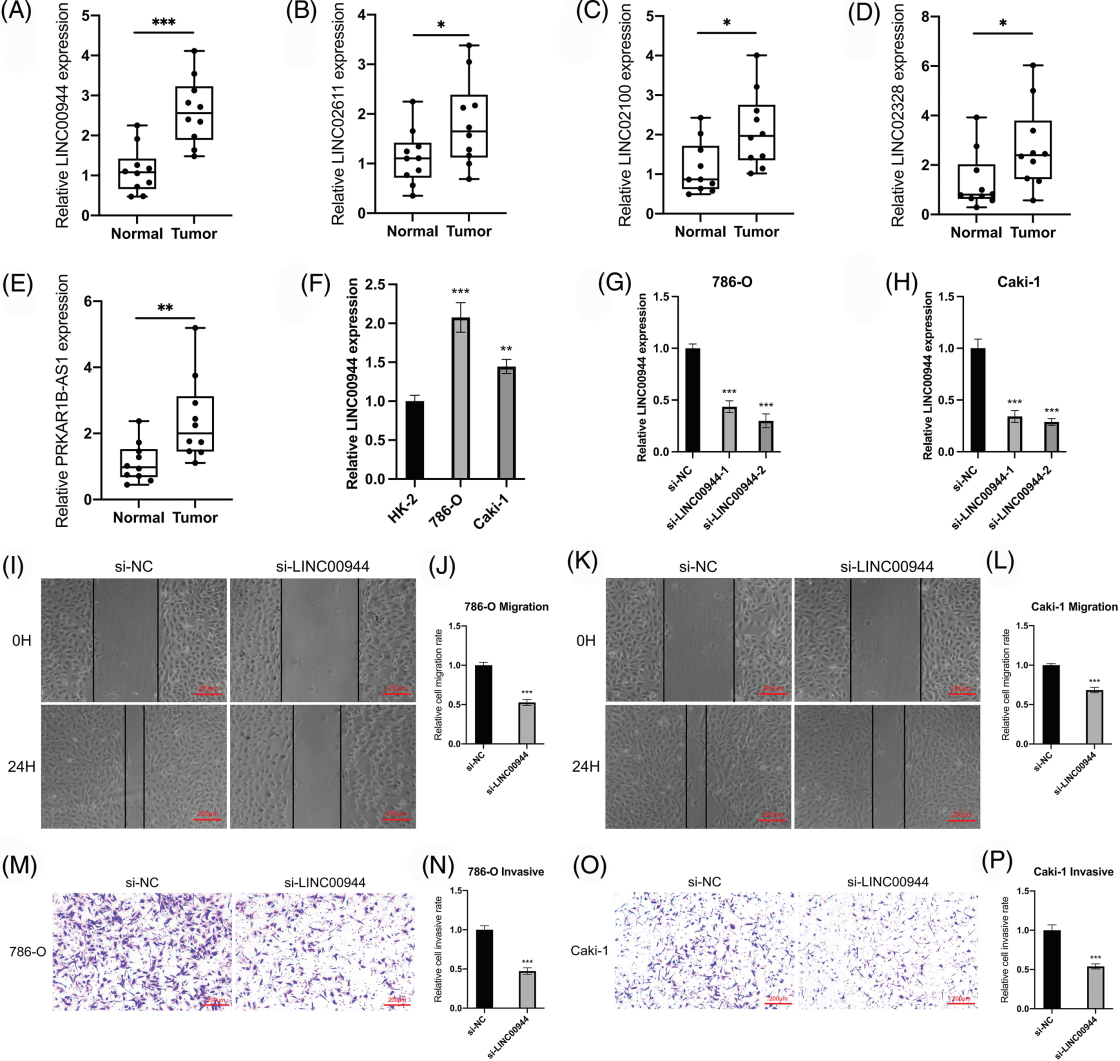
Validation of the model and exploration of biological characteristics. (A–E) Expression of model lncRNAs in 10 pairs of tissue specimens. (F) Expression of LINC00944 in different cell lines (n = 3). (G, H) Expression of LINC00944 after siRNAs transfection (n = 3). (I–L) Changes in migration ability of ccRCC cells after LINC00944 knockdown (n = 3). Scale bar = 200 μm. (M–P) Changes in invasion ability of ccRCC cells after LINC00944 knockdown (n = 3). Scale bar = 200 μm. *p < 0.05, **p < 0.01, and ***p < 0.001.

We verified the expression of the model-related lncRNAs in 10 pairs of tissue specimens collected from the clinic. LINC00944, LINC02611, PRKAR1B-AS1, LINC02328 and LINC02100 were expressed at higher levels in ccRCC tumor tissues compared to that in normal tissues (Figs. 11A–11E), which was consistent with the results of the TCGA-ccRCC cohort.

### LINC00944 and LINC02611 are oncogenes of ccRCC

**Figure 12:**
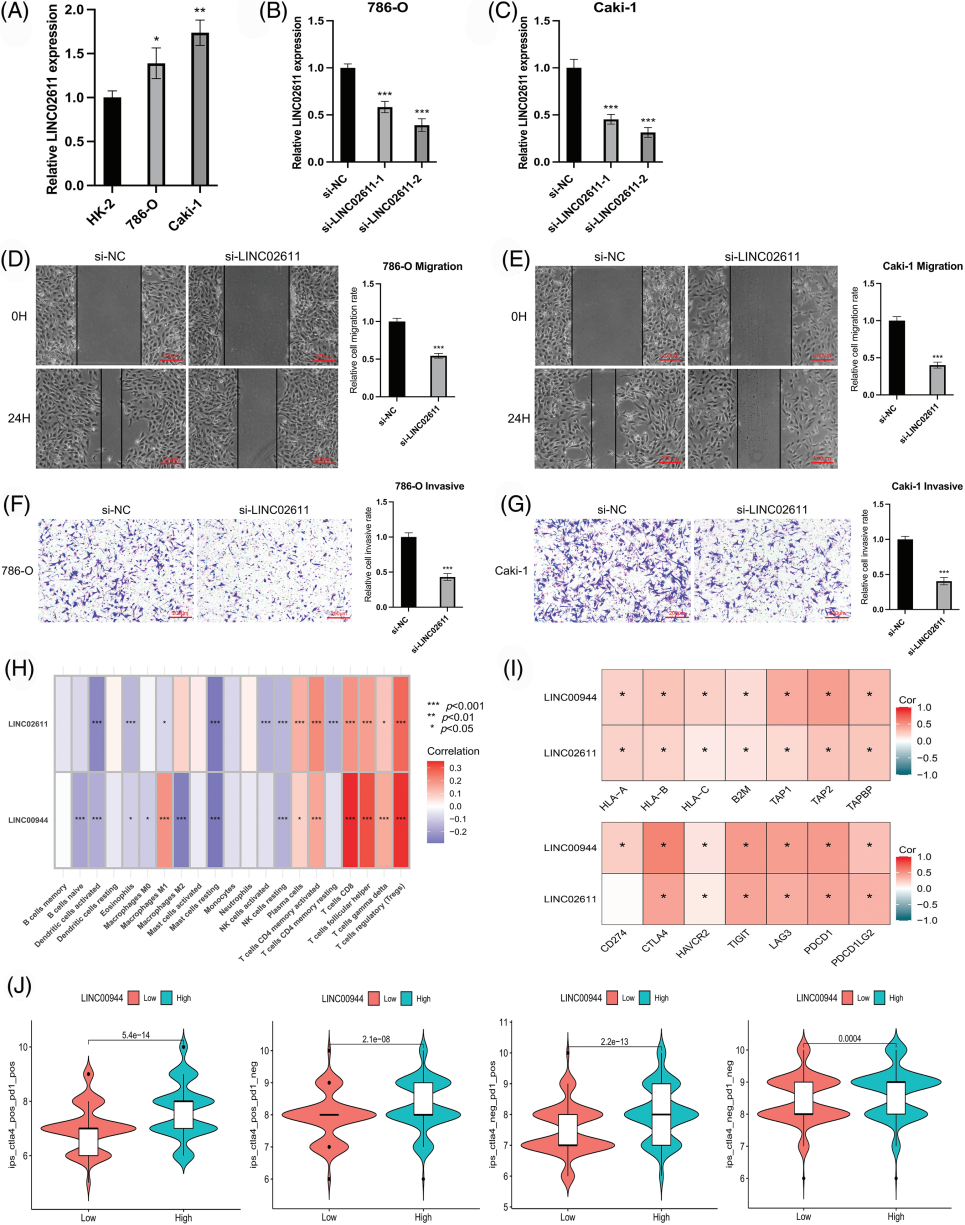
Exploration of the biological characteristics. (A) Expression of LINC02611 in different cell lines (n = 3). (B, C) Expression of LINC02611 after siRNAs transfection (n = 3). (D, E) Changes in migration ability of ccRCC cells after LINC02611 knockdown (n = 3). Scale bar = 200 μm. (F, G) Changes in invasion ability of ccRCC cells after LINC02611 knockdown (n = 3). Scale bar = 200 μm. (H) Correlation between model lncRNAs and immune cell infiltration. (I) Correlation between model lncRNAs and immune-related genes. (J) Differences in response between high and low LINC00944 expression groups when receiving combined anti-CTLA4 and anti-PD-1 therapy and when receiving anti-PD-1 or anti-CTLA4 therapy alone. *p < 0.05, **p < 0.01, ***p < 0.001.

We selected two model-related lncRNAs (LINC00944 and LINC02611) with the highest risk coefficients to further explore the biological role of these lncRNAs (Figs. 11F–11P; Figs. 12A–12G). Following qRT-PCR analysis, LINC00944 and LINC02611 were observed to be highly expressed in ccRCC cell lines, indicating their potential oncogenic function in ccRCC. Subsequently, siRNAs were used to knockdown the expressions of LINC00944 and LINC02611. Given the high knockdown efficiency, si-LINC00944-2 and si-LINC02611-2 were used in subsequent studies. The migration and invasion abilities of ccRCC cells were significantly reduced after LINC00944 or LINC02611 knockdown as evidenced by the scratch and Transwell assays. Thus, LINC00944 and LINC02611 could promote the progression of ccRCC by enhancing cell migration and invasion and can act as critical biomarkers.

**Figure S3:**
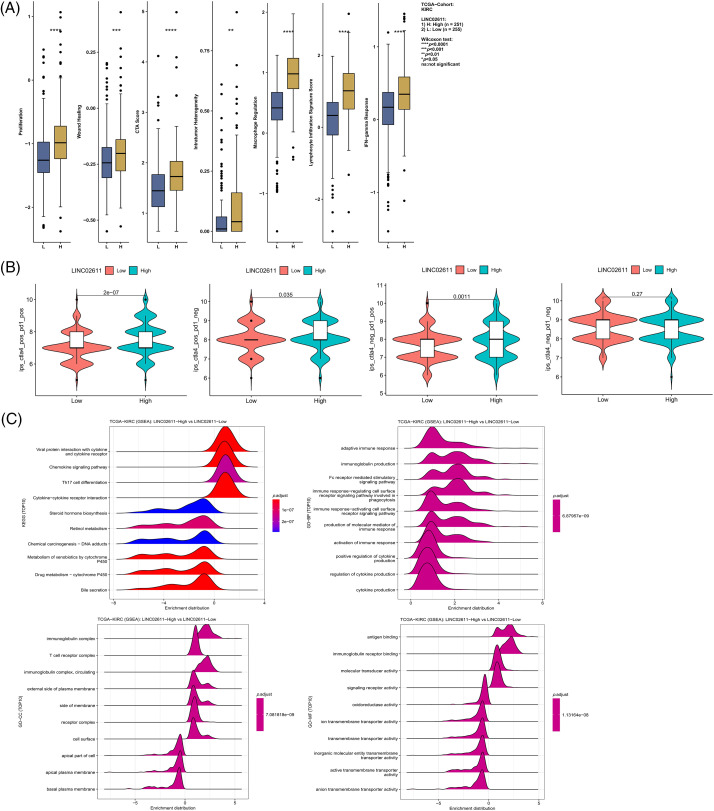
Differences in immune-related scores (A) and TCIA scores (B) between high and low expression groups of LINC02611. (C) Enrichment analysis of LINC02611.

To enrich our knowledge of these lncRNAs, we also explored their association with tumor immunity using the gene expression data of TCGA-ccRCC samples. LINC00944 exhibited a stronger correlation with immune infiltration and expressions of immune checkpoints and antigen-presenting genes than LINC02611 (Figs. 12H and 12I). Therefore, we focused on LINC00944 in the following analyses (Suppl. Fig. S2B; Fig. 12J). The relevant analyses for LINC02611 are shown in Suppl. Fig. S3. As shown in Suppl. Fig. S2B, the Intratumor Heterogeneity Immune Score, Macrophage Regulation, interferon-gamma (IFN-γ) Response, CTA Score, Proliferation and Wound Healing abilities were significantly high in the LINC00944 high expression group. Generally, tumor immunogenicity and antigen presentation are related to the patient’s response to immunotherapy [22]. Intratumor Heterogeneity and CTA scores can be used to evaluate tumor immunogenicity [23]. Accordingly, we preliminarily demonstrated that the expression of LINC00944 was related to immunotherapeutic responses. Subsequent analysis of TCIA scores further validated these results, suggesting that the high expression of LINC00944 was associated with a better response to immunotherapy (Fig. 12J).

**Figure 13:**
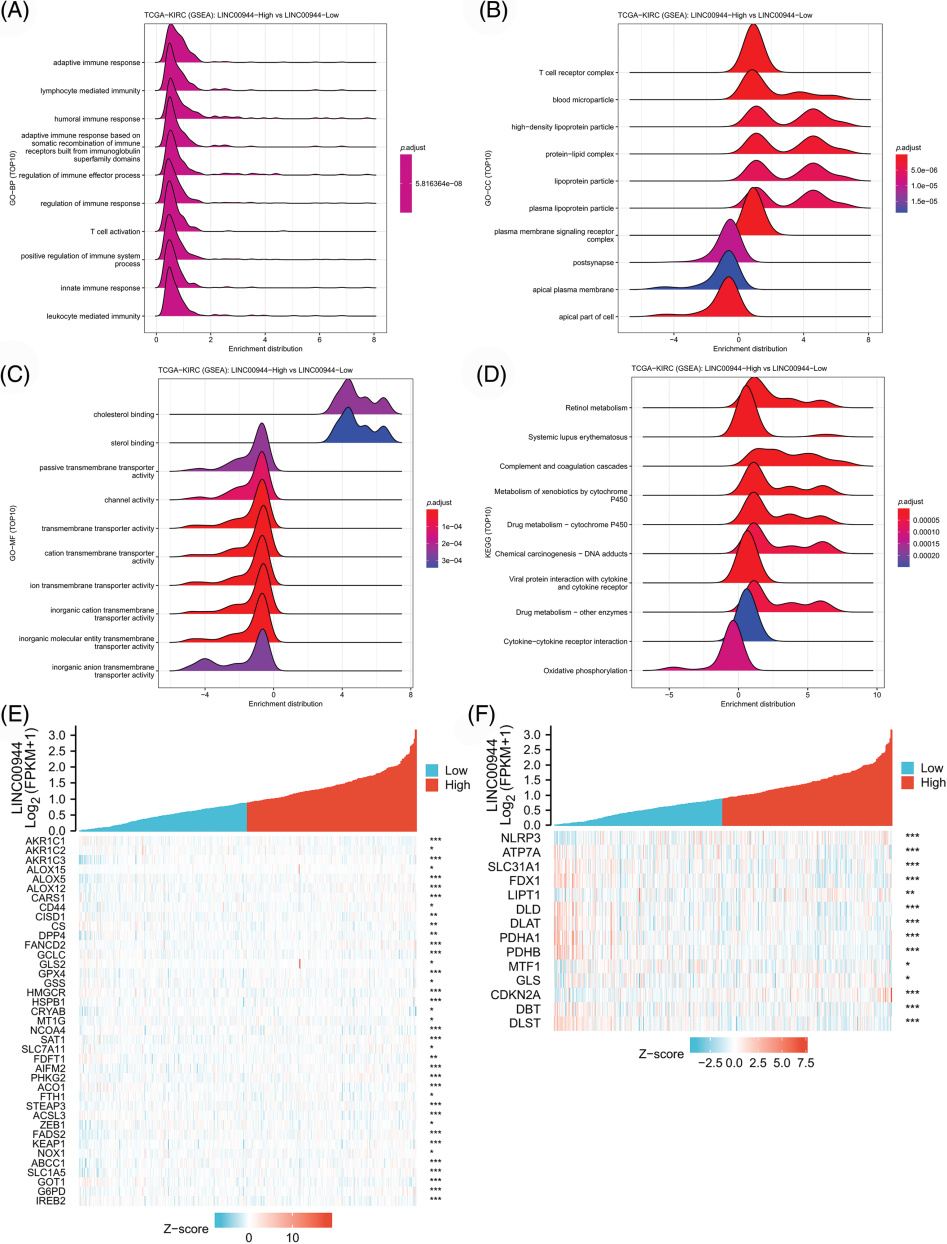
The biological value of LINC00944 in ccRCC. (A–D) Enrichment analysis of LINC00944. Association of LINC00944 expression with ferroptosis genes (E) and cuproptosis genes (F). *p < 0.05, **p < 0.01, and ***p < 0.001.

Enrichment analyses based on the TCGA-ccRCC cohort revealed that the high expression of LINC00944 was associated with the enrichment of cytokine−cytokine receptor interaction, T-cell activation and immune response regulation (Figs. 13A–13D). Conversely, the low expression of LINC00944 was associated with oxidative phosphorylation. Based on the observed interconnectivity between PCD, the correlation between LINC00944 and other forms of cell death was analyzed using the TCGA-ccRCC sample data. As shown in Figs. 13E and 13F, LINC00944 was significantly correlated with both ferroptosis and cuproptosis-related genes, demonstrating the interconnectedness of PCD. Additionally, it also demonstrated the importance of LINC00944 in PCD.

### Biological characteristics based on single-cell data

**Figure 14:**
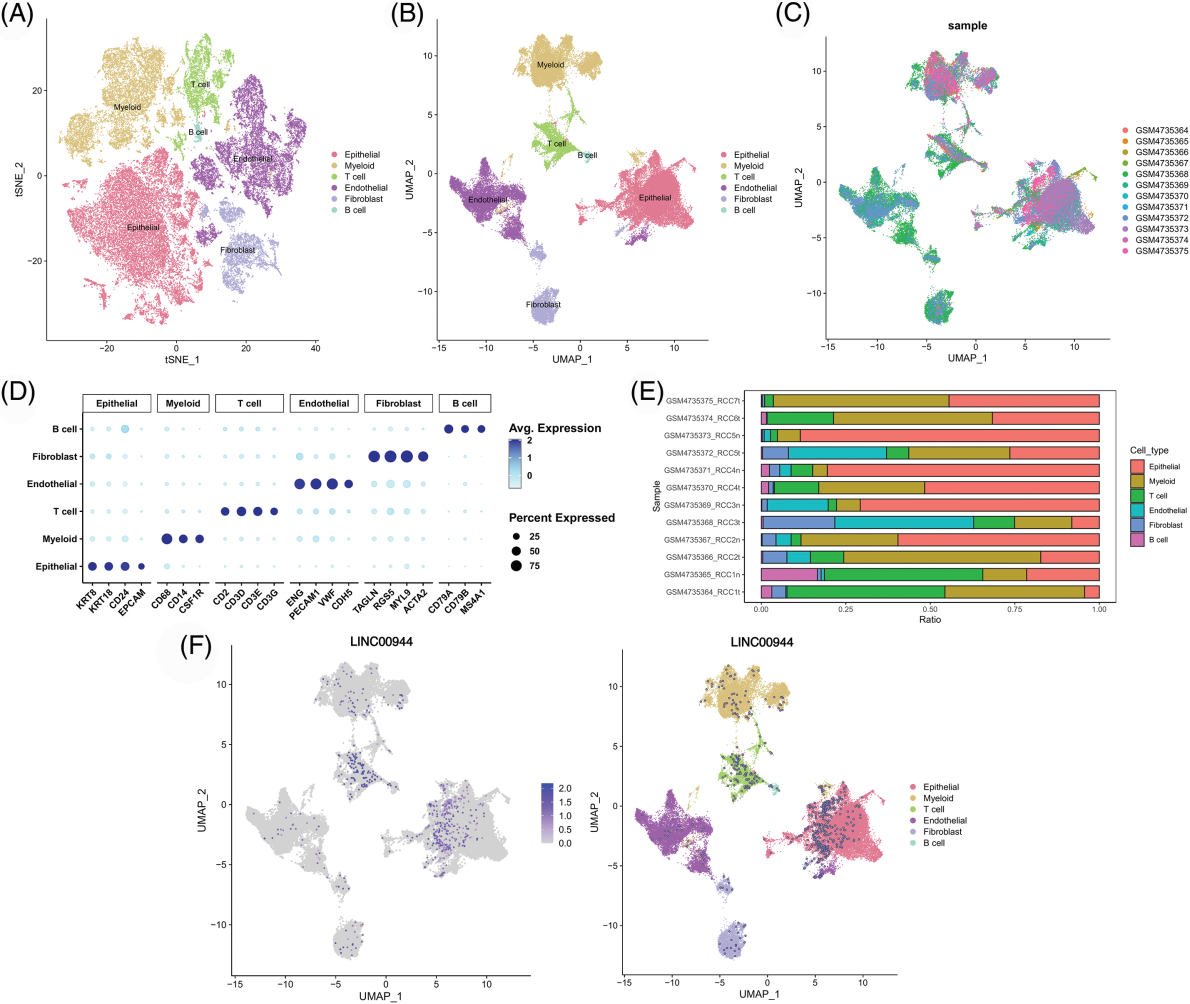
Single-cell atlas of ccRCC samples. TSNE (A) and UMAP (B) projections of different cell types. (C) The distribution of different samples. (D) Dot plot of the expression of marker genes in various cell types. (E) The cell composition of each sample. (F) UMAP projection of LINC00944.

**Figure S4:**
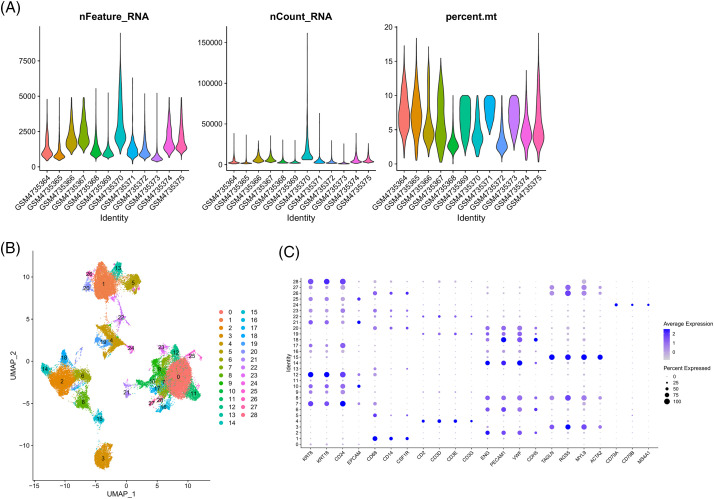
Single-cell analysis. (A) Quality control chart. (B) UMAP plot of all cell clusters. (C) Expression of gene markers in different clusters.

We classified the cells from the 12 samples obtained from GSE156632 into six types based on specific biomarkers (Figs. 14A–14C; Suppl. Fig. S4). The expression of cell-specific biomarkers in different cell types is shown in Fig. 14D. The cellular composition of each sample is shown in Fig. 14E. As shown in Fig. 14F, LINC00944 was mainly expressed in epithelial and T cells, suggesting a strong association between LINC00944 and immune cell infiltration apart from its significantly high expression in ccRCC tumor cells.

**Figure 15:**
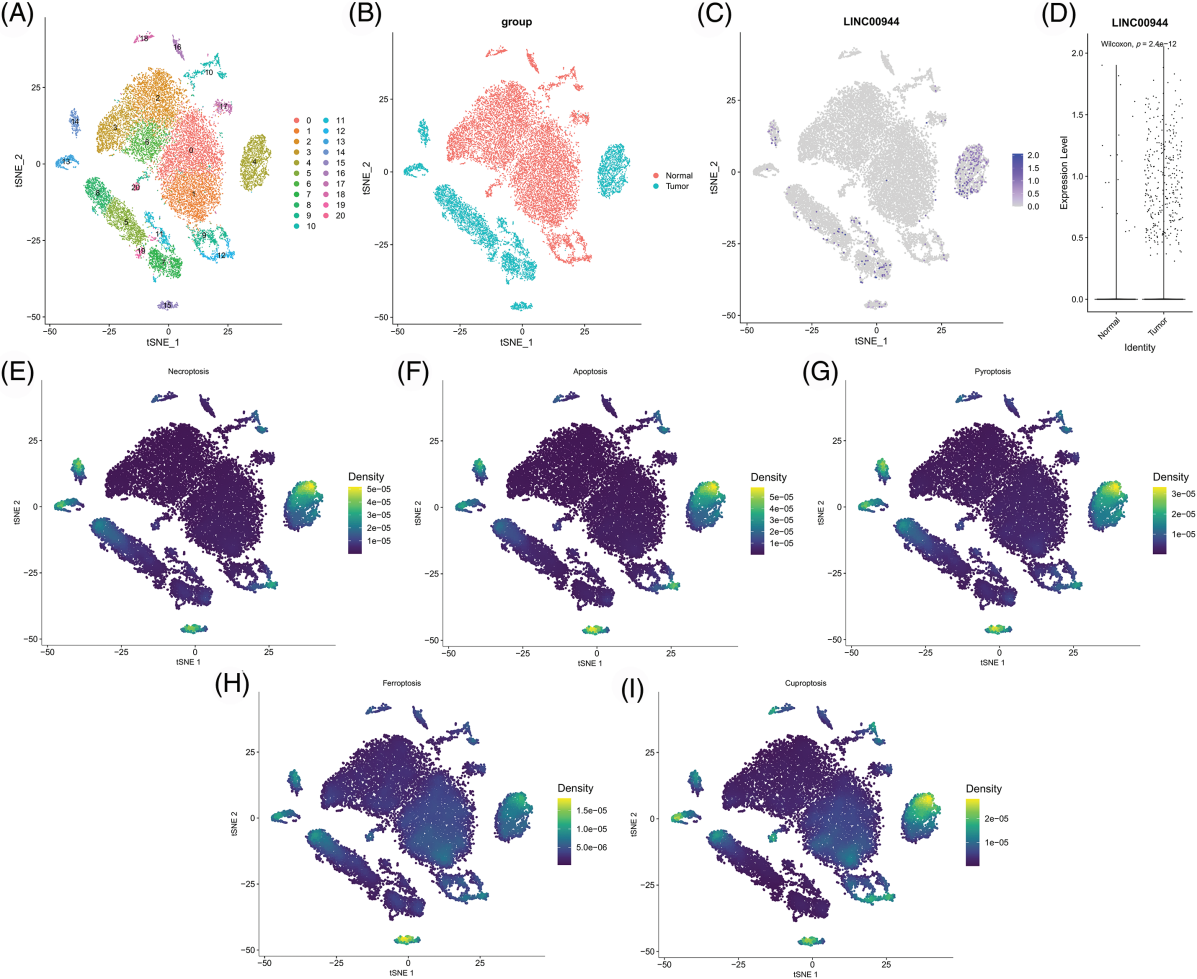
Re-clustering and analysis of epithelial cells. (A) TSNE plot of epithelial cell re-clustering. (B) TSNE plot of epithelial cells of different tissue origin. (C) TSNE projection of LINC00944 in epithelial cells. (D) Expression difference of LINC00944 among epithelial cells of different tissue origin. (E–I) TSNE projection of different PCD-related gene set scores in epithelial cells.

Subsequently, we extracted the epithelial cells for further analysis (Fig. 15A). The expression of LINC00944 in tumor epithelial cells was significantly higher than that in normal epithelial cells (Figs. 15B–15D), thereby further validating the above results. In addition, we calculated the scores of various PCD gene sets in epithelial cells. Notably, the epithelial cells with high PCD scores mapped similarly to those with high LINC00944 expression in the t-SNE plot (Figs. 15E–15I), which is consistent with previously reported results.

## Discussion

PANoptosis is a newly identified form of PCD and involves the activation of apoptosis, necroptosis, and pyroptosis. Since pyroptosis and necroptosis are both immunogenic cell death types, PANoptosis shows strong immunogenicity and can trigger immune responses and the activation of immune pathways [1]. lncRNAs, as an important factor influencing cancer progression, also showed a strong correlation with cancer immunity [24,25]. For example, LINC00152 is known to mediate the infiltration of CD8 T cells by interacting with EZH2, thereby affecting the progression of gastric cancer [26]. PANoptosis, lncRNAs, and tumor immunity are interrelated and exert differential effects on the occurrence and development of cancer. This study, however, is the first to combine these factors and investigate the impact of immune-associated PANoptosis lncRNAs on the immune microenvironment, cancer progression, and efficacy assessment in ccRCC.

By WGCNA analysis, we first confirmed the effect of PANoptosis on tumor immunity. We observed that PANoptosis-associated genes were strongly or weakly associated with different immune cells. In the subsequent consistent cluster analysis, the selected PANoptosis-related lncRNAs were significantly correlated with the progression, prognosis, high immune infiltration, immunosuppression status and activation of immune pathways of ccRCC. These findings revealed the critical function of PANoptosis-associated lncRNAs in ccRCC and provided a theoretical basis for further investigation of the mechanism.

Researchers have now developed many risk models for predicting the prognosis of patients with cancer [27–29]. Zhou et al. developed a novel glycosyltransferase-based prognostic model for predicting overall survival in hepatocellular carcinoma [29]. However, the predictive ability of the existing risk models is limited, and the development of better prognostic signatures remains vital. In this study, we constructed a risk model for predicting the prognosis of ccRCC based on immune-associated PANoptosis lncRNAs for the first time. Relevant evaluation and verification revealed that our risk model has high accuracy, reliability and applicability. Additionally, the construction of this model also demonstrated the influence of PANoptosis-related lncRNAs on ccRCC progression.

Currently, several biomarkers have been identified that predict patient response to immunotherapy, such as PD-L1, TMB (tumor mutational burden), GEP (gene expression profiles), TILs (tumor infiltration lymphocytes) and PD-L2 [30]. However, the high heterogeneity of ccRCC hinders the predictive capability of these biomarkers. Therefore, there remains a need to explore better biomarkers to evaluate the efficacy of immunotherapy. Based on previous studies and our analysis, high infiltration of CD8 T cells was associated with better benefits of immunotherapy compared to low CD8 T cell infiltration [20,31,32]. Accordingly, we selected CD8 T cell-associated PANoptosis lncRNAs to construct our risk model. The constructed model showed a strong correlation with immunotherapy efficacy, thereby providing a novel method to assess the response of ccRCC to immunotherapy.

Furthermore, the model-related lncRNAs LINC00944 and LINC02611 also play an oncogenic effect in ccRCC. The migration and invasion ability of ccRCC cells were significantly decreased after the knockdown of these lncRNAs. This suggests that these lncRNAs could promote the progression of ccRCC by affecting the migration of cancer cells. To enrich our understanding of these lncRNAs, we performed a series of analyses combining RNA transcriptome and single-cell data and selected LINC00944 as the study object. LINC00944 was associated with immune infiltration, immune-related pathway activity and PCD. T cell activation requires the assistance of antigen presentation machinery. Activated T cells can release several cytokines including IFN-γ, which upregulates genes related to antigen presentation and leads to high T cell aggregation [21,33]. In this study, the high expression of LINC00944 was significantly associated with both antigen presentation and IFN-γ response, highlighting the significance of LINC00944 in the activation and infiltration of T cells. Moreover, LINC00944 expression was correlated with the response to immunotherapy. Therefore, this lncRNA has the potential as a novel biomarker for assessing the efficacy of immunotherapy.

However, there are some limitations to our study. First, the predictive power of our risk model needs to be further validated in prospective studies. Second, our study of the biological properties of LINC00944 and LINC02611 is based on RNA transcriptome data, single-cell data and some cellular experiments. Therefore, further functional experiments and tissue validation are needed. Moreover, owing to the individual differences between patients with ccRCC, collecting and integrating more sample datasets can help us reassess the role of immune-related PANoptosis lncRNAs.

The effects of immune-related PANoptosis lncRNAs on ccRCC progression, prognosis, immune regulation and efficacy evaluation were elucidated herein. Based on these lncRNAs, we constructed a novel risk signature for predicting ccRCC prognosis and immunotherapy response. Furthermore, the mechanism of PANoptosis-related lncRNA in ccRCC was preliminarily explored using single-cell data. Overall, we identified LINC00944 and LINC02611, especially LINC00944, as promising prognostic biomarkers in ccRCC.

## Figures and Tables

**Table S1:** The list of necroptosis/apoptosis/pyroptosis-related genes

Necroptosis-related genes		Apoptosis-related genes		Pyroptosis-related genes	
FADD	BACH2	AIFM1	IRAK2	BAK1	PJVK
FAS	GATA3	AKT1	IRAK3	BAX	PLCG1
FASLG	MYCN	AKT2	IRAK4	CASP1	PRKACA
MLKL	ALK	AKT3	MAP3K14	CASP3	PYCARD
RIPK1	ATRX	APAF1	MYD88	CASP4	SCAF11
RIPK3	TERT	ATM	NFKB1	CASP5	TIRAP
TLR3	SLC39A7	BAD	NFKBIA	CHMP2A	TNF
TNF	SPATA2	BAX	NGF	CHMP2B	GZMA
TSC1	RNF31	BCL2	NTRK1	CHMP3	
TRIM11	IDH1	BCL2L1	PIK3CA	CHMP4A	
CASP8	IDH2	BID	PIK3CB	CHMP4B	
ZBP1	KLF9	BIRC2	PIK3CD	CHMP4C	
MAPK8	HDAC9	BIRC3	PIK3CG	CHMP6	
IPMK	HSP90AA1	CAPN1	PIK3R1	CHMP7	
ITPK1	LEF1	CAPN2	PIK3R2	CYCS	
SIRT3	BNIP3	CASP10	PIK3R3	ELANE	
MYC	CD40	CASP3	PIK3R5	GSDMD	
TNFRSF1A	BCL2L11	CASP6	PPP3CA	GSDME	
TNFSF10	EGFR	CASP7	PPP3CB	GZMB	
TNFRSF1B	DDX58	CASP8	PPP3CC	HMGB1	
TRAF2	TARDBP	CASP9	PPP3R1	IL18	
PANX1	APP	CFLAR	PPP3R2	IL1A	
OTULIN	TNFRSF21	CHP1	PRKACA	IL1B	
CYLD		CHP2	PRKACB	IRF1	
USP22		CHUK	PRKACG	IRF2	
MAP3K7		CSF2RB	PRKAR1A	TP53	
SQSTM1		CYCS	PRKAR1B	TP63	
STAT3		DFFA	PRKAR2A	AIM2	
DIABLO		DFFB	PRKAR2B	CASP6	
DNMT1		ENDOD1	PRKX	CASP8	
CFLAR		ENDOG	RELA	CASP9	
BRAF		EXOG	RIPK1	GPX4	
AXL		FADD	TNF	GSDMA	
ID1		FAS	TNFRSF10A	GSDMB	
CDKN2A		FASLG	TNFRSF10B	GSDMC	
HSPA4		IKBKB	TNFRSF10C	IL6	
BCL2		IKBKG	TNFRSF10D	NLRC4	
STUB1		IL1A	TNFRSF1A	NLRP1	
FLT3		IL1B	TNFSF10	NLRP2	
HAT1		IL1R1	TP53	NLRP3	
SIRT2		IL1RAP	TRADD	NLRP6	
SIRT1		IL3	TRAF2	NLRP7	
PLK1		IL3RA	XIAP	NOD1	
MPG		IRAK1		NOD2	

**Table S2:** The primers used for qRT-PCR

lncRNA	Primer	Sequence
LINC00944	Forward	AGACGCACATCAGGAAGACAG
LINC00944	Reverse	TGAGTTACAGGGACCGAAGC
LINC02611	Forward	CTTGTGTGCATTTCCTGCCT
LINC02611	Reverse	TGAAACTGCCTTGCCTAAAGC
PRKAR1B-AS1	Forward	TCCTCGAGACACGACAAGTTAG
PRKAR1B-AS1	Reverse	ACAGATGTGGAAGAGACAAACC
LINC02328	Forward	CAGTGGGACTGCTGAGTTGA
LINC02328	Reverse	TGCAAGTCCAAAGGCAAACAG
LINC02100	Forward	GGTCCATGGCTCCATTCCTT
LINC02100	Reverse	GGATGGGCCCCTAGAAATGA
GAPDH	Forward	GAGAAGGCTGGGGCTCATTT
GAPDH	Reverse	TAAGCAGTTGGTGGTGCAGG

**Table S3:** The sequence of the siRNAs

siRNA	Primer	Sequence
si-LINC00944-1	sense	GCUGAGCAGGAACAACUAUTT
si-LINC00944-1	antisense	AUAGUUGUUCCUGCUCAGCTT
si-LINC00944-2	sense	CCCGGAAACAUCAUCUCAUTT
si-LINC00944-2	antisense	AUGAGAUGAUGUUUCCGGGTT
si-LINC02611-1	sense	GGAUCUAAAGCUGGUGGAUTT
si-LINC02611-1	antisense	AUCCACCAGCUUUAGAUCCTT
si-LINC02611-2	sense	GCAUGAAAGCCUGCCAUUUTT
si-LINC02611-2	antisense	AAAUGGCAGGCUUUCAUGCTT
si-NC	sense	GGGUGGAAUUCCAGAACAATT
si-NC	antisense	UUGUUCUGGAAUUCCACCCTT

## Data Availability

The original datasets in this study were taken from online public databases. The details are presented in the article/Supplementary Materials.
